# EHMTI-0194. The impact of dizziness in tertiary care migraine patients

**DOI:** 10.1186/1129-2377-15-S1-D61

**Published:** 2014-09-18

**Authors:** J Sugrue, E Thomkins, M Ruttledge

**Affiliations:** 1 Headache Clinic, Beaumont Hospital, Dublin 9, Ireland

## Introduction

The association between dizziness and migraine has been proven. There is no data available on the relationship between dizziness and impact or disability scores in tertiary clinic migraine patients.

## Aims

1. Outline the incidence of dizziness in patients with migraine.

2. Compare Headache Impact Test (HIT-6) scores in patients with migraine and dizziness to those with migraine without dizziness.

## Methods

A questionnaire design study collected demographic data, headache characteristics and features, healthcare utilisation, and outcome measures in patients attending a tertiary headache clinic over a six week period. One aspect of questioning related to dizziness as an associated feature. Analysis was carried out only on those who fulfilled the International Headache Society (IHS) classification for episodic or chronic migraine. Post-hoc analysis examined the differences in HIT-6 scores in subgroups using a Kruskall-Wallis test.

## Results

Of the 61 respondents, analysis was carried out on the 51 who fulfilled the IHS migraine criteria. Of these, 84% were female with a mean age of 42.1 years (SD 14.5). Mean headache days per month was 13.4 (SD 10.5), while 45% had chronic migraine. Dizziness was experienced by 92% of patients with migraine, and those with dizziness had significantly higher HIT-6 scores (p=0.04).

## Conclusion

Patients with migraine and dizziness experience a more severe impact on their daily lives than those without dizziness. This preliminary work has implications for future research which should be directed to the assessment and management of dizziness in migraine.

No conflict of interest.

